# Identification of Stabilization of Malvid Anthocyanins and Antioxidant Stress Activation via the AMPK/SIRT1 Signaling Pathway

**DOI:** 10.1155/2021/9934646

**Published:** 2021-08-26

**Authors:** Fei Zheng, Hai Xue, Bi-Xiang Wang, Man-Yu Wu, Dong-Xia Chen, Hao Yue, Lian-Kui Wen, Yang He

**Affiliations:** ^1^College of Food Science and Engineering, Jilin Agricultural University, Changchun 130118, Jilin, China; ^2^Jilin Ginseng Academy, Changchun University of Chinese Medicine, Changchun 130117, Jilin, China; ^3^School of Tourism, Jilin Business and Technology College, Changchun 130507, Jilin, China

## Abstract

*Vitis amurensis* Rupr. “Beibinghong” is abundant in anthocyanins, including malvidin (Mv), malvidin-3-glucoside (Mv3G), and malvidin-3,5-diglucoside (Mv35 G). Anthocyanins offer nutritional and pharmacological effects, but their stability is poor. Interaction of malvid anthocyanins with caffeic acid through ultrahigh pressure technology produces stable anthocyanin derivatives. This study aims to identify the structure of stable mallow-like anthocyanins and to determine the effect of these stable anthocyanins on human umbilical vein endothelial cells (HUVECs) with H_2_O_2_-induced oxidative damage and the signaling pathway involved. The products of malvid anthocyanins and caffeic acid bonding were identified and analyzed using ultra-high performance liquid chromatography-quadrupole-Orbitrap mass spectrometry (UPLC-Q-Orbitrap MS/MS). The bonding products were malvidin-3-O-guaiacol (Mv3C), malvidin-3-O-(6″-O-caffeoyl)-glucoside (Mv3CG), and malvidin-3-O-(6″-O-caffeoyl)-5-diglucoside (Mv3C5G). An oxidative stress injury model in HUVECs was established using H_2_O_2_ and treated with Mv, Mv3G, Mv35 G, Mv3C, Mv3CG, and Mv3C5G at different concentrations (10, 50, and 100 *μ*mol/L). Results showed that the above compound concentrations can significantly increase cell proliferation rate and reduce intracellular reactive oxygen species at 100 *μ*mol/L. The effects of the most active products Mv and Mv3C on the AMP-activated protein (AMPK)/silencing information regulator-1 (SIRT1) pathway were analyzed. Results showed that Mv and Mv3C significantly increased SOD activity in the cells and significantly upregulated the expression of SIRT1 mRNA, SIRT1, and *p*-AMPK protein. However, they did not significantly change the expression of AMPK protein. After the silent intervention of siRNA in SIRT1 gene expression, the upregulation of SIRT1 and *p*-AMPK protein by Mv and Mv3C was significantly inhibited. These results indicate that stabilization malvid anthocyanins exerts an antioxidant activity via the AMPK/SIRT1 signaling pathway.

## 1. Introduction

Oxidative stress injury is an important risk factor for human aging, subhealth, and various diseases, such as tumors, diabetes, cardiovascular and cerebrovascular diseases, Alzheimer's disease, and neuropathy. The body produces a large number of reactive oxygen species (ROS) during oxidative stress. In addition to direct damage to cells, ROS can also activate different oxidative stress pathways, consequently affecting the degree of tissue or cell damage. Oxidative stress can directly or indirectly activate AMP-activated protein (AMPK) through ROS or reactive nitrogen species. AMPK is a key protein involved in multiple signal transduction pathways, and AMPK signaling pathway imbalance is closely related to oxidative stress [1–4]. AMPK, through its interaction with mTOR and SIRTuins, is a key regulator of aging. Silencing information regulator-1 (SIRT1) is a NAD^+^-dependent deacetylase widely present in mammals and is involved in the regulation of cell proliferation, aging, and death, inflammation, and metabolic regulation [5, 6]. Recent studies have shown that the AMPK/SIRT1 signaling pathway is closely related to oxidative stress, aging, diabetes, and tumors [7–10].

Natural anthocyanins have significant antioxidant activity and can eliminate free radicals in the body. Our previous study found that the anthocyanin content of *Vitis amurensis* Rupr. “Beibinghong” in the Changbai Mountains of China is about 180 mg/(100 g·FW), and the malvid anthocyanin content is the highest, accounting for about 60% of the total anthocyanin content, including malvidin, malvidin-3-O-glucoside, and malvidin-3,5-O-diglucoside [11]. Malvid anthocyanins have various physiologically active functions, such as antioxidant, antiaging, anticardiovascular disease, and anticancer, with a potential role in preventing various chronic diseases in humans [12].

However, the poor stability of natural anthocyanins limits their application. Ultrahigh pressure can promote the mutual color of anthocyanins and organic acids in the grapevine in a short time, with a remarkable stabilizing effect [13]. A previous study optimized the ultrahigh pressure bonding of malvid anthocyanins and caffeic acid by using the response surface method to achieve stabilization of malvid anthocyanins with a color increase rate of 42.986% ± 0.09%; after heat processing at 100°C for 2 h or placing for 20 days under room temperature sunlight, it can still achieve about 50% preservation rate and good photothermal stability. The results of in vitro antioxidant activity showed that the stable anthocyanins at 20 *μ*g/mL are effective against DPPH, and the free radical scavenging rate of ABTS is >90% [13].

This study aimed to evaluate whether the stabilization of malvid anthocyanins is capable of activating antioxidant stress. An in vitro oxidative stress model was established using H_2_O_2_-injured human umbilical vein endothelial cells (HUVECs) to observe the protective effect of stabilization of malvid anthocyanins on the oxidative stress damage of HUVECs and to explore its mechanism of action. This study lays a foundation for the efficient use of anthocyanins and serves as a theoretical basis for the in-depth development of mountain grape resources in the Changbai Mountains.

## 2. Materials and Methods

### 2.1. Materials and Reagents

Malvidin, malvidin-3-O-glucoside, and malvidin-3,5-O-diglucoside were purchased from ChromaDex (Irvine, USA). Caffeic acid and formic acid were purchased from Aladdin. Chromatographic-grade methanol was purchased from Thermo Fisher (Waltham, USA). Dulbecco's modified Eagle's medium (DMEM), penicillin-streptomycin (P/S), and fetal bovine serum (FBS) were bought from Gibco Life Technologies (Grand Island, NY, USA). CCK-8 cell proliferation and toxicity detection kit were procured from Dojindo Chemical Research Institute (Kyushu, Japan). Cell ROS detection kit and SOD kit were purchased from Shanghai Beyotime Biological Co., Ltd. (Shanghai, China).

### 2.2. Extraction and Separation of Malvid Anthocyanins

*V*. *amurensis* Rupr. “Beibinghong” were collected from Liuhe County (Tonghua Wine Institute, Tonghua, China) in October 2017. Liuhe County is located in the east longitude 125°17′-126°35′ and latitude 41°54′-24°35′, with an annual average temperature of 4.4–5.5°C, annual minimum temperature of -36.2°C, frost-free period of 126–140 d, activity accumulated temperature of 2800.2°C, annual precipitation of 700–900 mm, and sunshine for 2550 h. *V*. *amurensis* Rupr. “Beibinghong” were collected with the stems removed and then beaten for use. On the basis of previous studies [14], the high-voltage pulsed electric field method was used for extraction, D101 for macroporous resin purification, and high-performance liquid phase for monomer preparation. The obtained anthocyanins included malvidin (Mv), malvidin-3-glucoside (Mv3G), and malvidin-3,5-diglucoside (Mv35 G). *V*. *amurensis* Rupr. “Beibinghong” (100 g) contained 108 mg of malvid anthocyanins (40.8% Mv35 G, 48.6% Mv3G, and 10.6% Mv).

### 2.3. Ultrahigh Pressure-Assisted Preparation of Caffeic Acid-Bonded Malvid Anthocyanins

The ultrahigh pressure bonding method optimized using the response surface method was used [13]. Caffeic acid was dissolved in a small amount of absolute ethanol. The mallow anthocyanin-to-caffeic acid mass ratio of 1 : 3.6 was added, and then the solution was diluted with dibasic sodium phosphate–citric acid buffer solution at pH 3.0. After 1 h of water bath at 60°C, ultrahigh pressure bonding was used to achieve the pressure of 300 MPa for 5 min of holding time. The solution produced after ultrahigh pressure bonding was purified, collected, frozen, and then dried for further use.

### 2.4. Liquid Chromatography-Mass Spectrometry

In our previous work [13], a combination of high-performance liquid chromatography, infrared spectroscopy, and liquid chromatography-mass spectrometry was used to identify the bonded compounds initially. In this experiment, UPLC-Q-Orbitrap MS/MS high-resolution mass spectrometry was performed to verify the bonded compounds.

Liquid chromatography conditions were as follows: Thermo Syncronis C18 chromatographic column (100 mm × 3 mm, 1.7 *μ*m), gradient elution, and a mobile phase of 0.1% formic acid aqueous solution (A) and acetonitrile (B). The gradient elution was as follows: 0–2 min, 8%–10% B; 2–11 min, 10%–10% B; 11–19 min, 10%–15% B; 19–24 min, 15%–20% B; 24–27 min, 20%–25% B; 27–33 min, 25%–35% B; 33–36 min, 35%–45% B; 36–38 min, 45%–55% B; and 38–45 min, 55%–80% B. The column temperature was 30°C, flow velocity was 0. 2 mL·min^−1^, and injection volume was 10 *μ*L. Mass spectrometry conditions were as follows: electrospray positive ion scanning mode, drying air temperature of 350°C, atomizing gas flow rate of 35 arb, auxiliary gas flow rate of 10 arb, and quality scan range of *m/z* 50–1000.

### 2.5. Cell Culture

The human umbilical vein endothelial cell line (HUVEC) was purchased from Wuhan Procell Life Science and Technology Co., Ltd. (Wuhan, China). The primary HUVECs were placed in culture flasks in a 5% CO_2_ incubator at 37°C. After the growth was stable, the culture growth was continued until cell passage, and the cells were frozen. Four to nine generations of cells with good growth status were selected for the experiment.

### 2.6. Cell Transfection

The HUVECs in the logarithmic growth phase were inoculated in a 96-well cell culture plate by 5 × 10^5^ per well at 37°C in a 5% CO_2_ incubator overnight. At 2 h before transfection, the medium was replaced by a serum-free DMEM medium. During transfection, 10 *μ*L of siRNA plasmid (20 *μ*mol/L) was diluted with 100 *μ*L of serum-free Opti-MEM, mixed well, and then allowed to stand at room temperature for 5 min. Then, 5 *μ*L of Lipofectamine 2000 liposomes were diluted with 100 *μ*L of Opti-MEM and allowed to stand at room temperature for 5 min. Lipofectamine 2000 liposomes were mixed with the siRNA plasmid diluent and then allowed to stand at room temperature for 20 min. The incubated mixture was added to the six-pore plate without serum, mixed well, and then placed in a 5% CO_2_ incubator at 37°C for 6 h to aspirate the mixture and change into DMEM medium. The culture was continued for 48 h at 37°C in a 5% CO_2_ incubator.

### 2.7. CCK-8 Detection of Cell Proliferation Rate

HUVECs in the logarithmic growth phase were inoculated in a 96-well cell culture plate by 5 × 10^3^ per well. The cells in each well were added with 200 *μ*L of culture medium, with three parallel samples, and then cultured in a 5% CO_2_ incubator at 37°C for 24 h. The blank, model, and test groups of different concentrations (10, 50, and 100 *μ*mol/L) were treated with caffeic acid, Mv, malvidin-3-O-guaiacol (Mv3C), Mv3G, malvidin-3-O-(6″-O-caffeoyl)-glucoside (Mv3CG), Mv35 G, and malvidin-3-O-(6″-O-caffeoyl)-5-diglucoside (Mv3C5G) for 24 h. Then, 20 *μ*L of CCK-8 solution was added to each well and cultured at 37°C for 4 h. A microplate reader was used to measure the absorbance of each well (*OD* 450).

(1) $$\begin{array}{c} \text{Cell proliferation rate}\left(\%\right)=\left(\frac{{\text{OD}}_{\text{sample group}}}{{\text{OD}}_{\text{control group}}}\right)\times 100, \end{array}$$

(2) $$\begin{array}{c} \text{cell inhibition rate}\left(\%\right)=1-\left(\frac{{\text{OD}}_{\text{sample group}}}{{\text{OD}}_{\text{control group}}}\right)\times 100, \end{array}$$

where OD_sample group_ is the absorbance value of the test group and OD_control group_ is the absorbance value of the control group.

### 2.8. Determination of Intracellular ROS Level and SOD Activity

HUVECs in the logarithmic growth phase were inoculated in a 96-well cell culture plate with 5 × 10^3^ per well. The cells in each well were added with 200 *μ*L of culture medium and then cultured in a 5% CO_2_ incubator at 37°C for 24 h. The blank, model, and test groups of different concentrations (10, 50, and 100 *μ*mol/L) were treated with caffeic acid, Mv, Mv3C, Mv3G, Mv3CG, Mv35 G, and Mv3C5G for 24 h, and then the cells were washed once with PBS. In accordance with the kit instructions, a serum-free medium was used to dilute DCFH-DA by 1 : 1000 to make the final concentration of 10 *μ*mol. The cells were added to 200 *μ*L of diluted DCFH, incubated at 37°C for 20 min, and then mixed well every 3 min. Then, the cells were washed with serum-free medium twice, resuspended with PBS, and subjected to fluorescence detection with a microplate reader (excitation wavelength at 480 nm, emission wavelength at 525 nm).

(3) $$\begin{array}{c} \text{Ratio}\left(R\right)=\frac{A_{\text{sample group}}}{A_{\text{control group}}}, \end{array}$$

where *A*_sample group_ is the absorbance value of the test group and *A*_control group_ is the absorbance value of the control group.

HUVECs in the logarithmic growth phase were treated in each group and then collected using a cell scraper. The cells were lysed by adding 200 *μ*L of PBS and then disrupted in a 4°C centrifuge at 12000 r/min for 10 min. The supernatant was taken and tested for activity using the SOD kit in accordance with the manufacturer's instructions.

### 2.9. H_2_O_2_-Induced Oxidative Damage Model in HUVECs

The HUVECs were treated with H_2_O_2_ at 100, 200, 300, 500, 750, and 1000 *μ*mol/L for different time periods (6, 12, 24, and 48 h), and then the cell inhibition rate was detected using the CCK-8 method. Three replicate wells were set in each group, and the results were averaged to calculate the inhibition rate (%) of cell proliferation by the different concentrations of H_2_O_2_. In subsequent experiments, the level of intracellular ROS was measured using 50% (IC_50_) H_2_O_2_ as the H_2_O_2_ concentration.

### 2.10. Real-Time Quantitative PCR Detection of SIRT1 mRNA Expression

The total RNA of the cells in each treatment group was extracted using the RNA rapid extraction kit. RNA was reversely transcribed to cDNA using the reverse transcription kit for PCR amplification. The SIRT1 amplification primer was purchased from Sangon Biotech (Shanghai, China): Primer Forward, 5′-CAAAGGAGCAGATTAGTAGG-3′; Reverse, 5′-CTGCCACAAGAACTAGAGGA-3′. Adding internal reference genes, 2^-△△Ct^ was used to analyze and calculate the relative expression of SIRT1 mRNA.

### 2.11. Western Blot Analysis

After the drug action, the cells were collected to add the lysate containing PMSF to extract the total protein and measure the protein concentration using the BCA kit. The cells were heated for protein denaturation, loaded with 25 *μ*g of protein or SDS-PAGE gel electrophoresis, transferred onto PVDF membranes, and then stored at room temperature for 2 h with 5% skim milk. Primary antibodies *β*-actin (1 : 200), AMPK (1 : 1000), *p*-AMPK (1 : 1000), and SIRT1 (1 : 1000) were added and then incubated at 4°C overnight. After adding a secondary antibody and incubating at room temperature for 2 h, the color of the ECL solution was developed and the image was scanned. The ratio of the target protein to the corresponding net optical density of the *β*-actin band indicates the protein expression level of each group.

### 2.12. Statistical Analysis of Data

Data were analyzed using the SPSS statistical software system. Experimental data were expressed as mean ± standard deviation ((*x* ± *s*)). Comparison between multiple groups was performed by ANOVA, and comparison between two groups was performed by *q* test. Significance and extreme significance were indicated by *p* < 0.05 and *p* < 0.01, respectively. Origin 8.0 software was used for drawing.

## 3. Results

### 3.1. Structural Analysis of Stable Malvid Compounds

**Figure 1:**
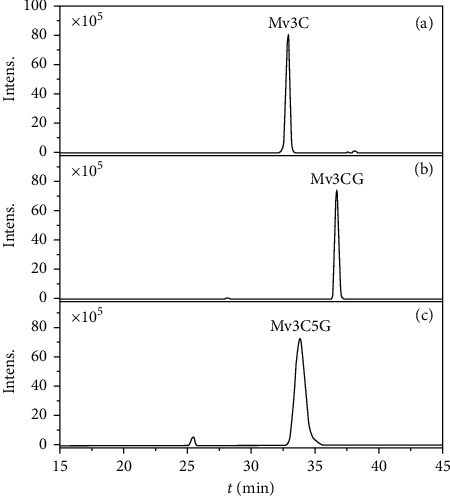
Total ion chromatogram of the bonded products.

**Figure 2:**
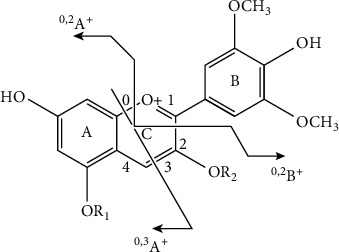
Characteristic product ions of anthocyanidins.

The chemical constituents of the bonded products were identified by comparing their accurate masses and characteristic fragment ions via UPLC-Q-Orbitrap MS/MS. The base peak chromatogram profiles of the bonded products are shown in Figure 1. On the basis of previous studies [15, 16], the characteristic product ions of anthocyanin fragmentation in tandem mass spectrometry are shown in Figure 2, and the MS/MS results of the bonded products M1, M2, and M3 are shown in Table 1. M1 molecular ion *m/z* 439.10351[M+H]^+^ and fragment ions *m/z* 331.12247 and 150.33725 were observed using secondary tandem mass spectrometry. The fragment ion *m/z* 331 was produced by losing one molecule of *o*-hydroxyphenol (108), and *m/z* 150 was the ^0,2^A^＋•^ radical positive ion caused by breaking the 0/2 position C-C bond of the anthocyanin C-ring. In consideration of relevant reports [17–20], M1 was determined as malvidin-3-O-guaiacol (Mv3C). A molecular ion peak *m/z* 655.16632 [M+H]^+^ was observed in M2, and fragment ions *m/z* 493.17451, 331.14216, and 150.38797 were found in the secondary tandem mass spectra. *m/z* 493 and 331 were produced by the parent ion losing one molecule of caffeoyl group (162) and one molecule of glucose residue (162) successively. In consideration of relevant reports [21–24], M2 was identified as Mv3CG. A molecular ion peak *m/z* 817.21914[M+H]^+^ was found in M3, and fragment ions *m/z* 655.34326, 493.13761, 331.12026, 282.07894, 150.3890, and 121.02881 were detected in the secondary tandem mass spectra. Fragment ions *m/z* 655, 493, and 331 were formed by the parent ion losing two molecules of glucose residues and one molecule of caffeoyl. *m/z* 282.07894 and 121.02881 were found in the fragment ions. According to literature [25], *m/z* 282.07894 may be the ^0,3^A^＋•^ free radical positive ions caused by the breaking of C-C bond on the 0/3 position of anthocyanin C-ring, *m/z* 121.02881 is caused by the fragment ion *m/z* 282.07894 losing one molecular mass residue of 162 glucose, and the caffeoyl group may be linked to the glucoside at position 3. On the basis of analysis of relevant literature [26, 27], M3 was deduced as Mv3C5G.

### 3.2. H_2_O_2_-Induced Oxidative Damage Model of HUVECs

The half inhibitory concentration IC_50_ of H_2_O_2_-induced oxidative stress injury in HUVECs gradually decreased in a concentration- and time-dependent manner (Table 2). When the concentration of the H_2_O_2_ solution was 300 *μ*mol/L, the ROS ratio in the cells began to show a significant difference compared with that in the blank group (*p* < 0.05). When cultured for 24 and 48 h, the ROS ratio in the cells was significantly different from that in the blank group (*p* < 0.01). At 24 h, the ROS ratio was 2.56, and the cell damage was obvious and stable (Table 3). Therefore, the concentration of 300 *μ*mol/L H_2_O_2_ was chosen to induce HUVECs for 24 h.

### 3.3. Protective Effect of Stabilization of Malvid Anthocyanins on Cells with Oxidative Damage

**Figure 3:**
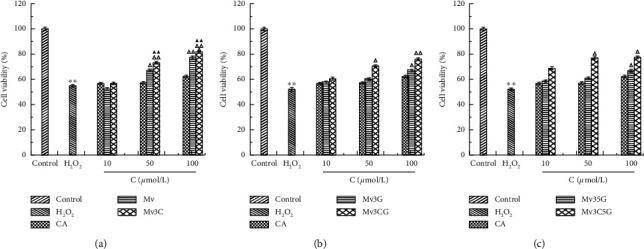
Effects of different concentrations of Mv and Mv3C (a); Mv3G and Mv3CG (b); and Mv35 G and Mv3C5G (c) on the appreciation rate of HUVEC cells.  ^*∗*^*p* < 0.05 and  ^*∗∗*^*p* < 0.01 compared to control; Δ*p* < 0.05 and ΔΔ*p* < 0.01 compared to H_2_O_2_; ▲*p* < 0.05 and, ▲▲*p* < 0.01 compared to malvid anthocyanins.

**Figure 4:**
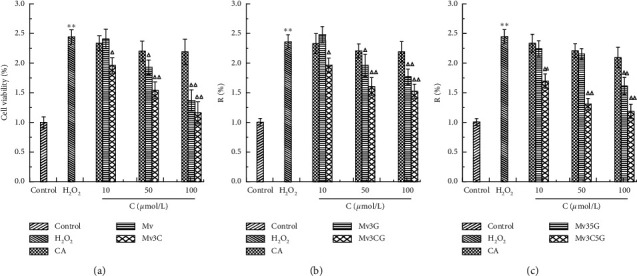
Effects of different concentrations of Mv and Mv3C (a); Mv3G and Mv3CG (b); and Mv35 G and Mv3C5G (c) on level of ROS of HUVEC cells. ^*∗*^*p* < 0.05 and ^*∗∗*^*p* < 0.01 compared to control; Δ*p* < 0.05 and ΔΔ*p* < 0.01 compared to H_2_O_2_; ▲*p* < 0.05 and ▲▲*p* < 0.01 compared to malvid anthocyanins.

The results of CCK8 detection and measurement of intracellular ROS levels are shown in Figures 3 and 4. The cell proliferation rate of the H_2_O_2_ oxidative damage model group was 0.54 times that of the blank control group, indicating successful modeling. At the same time, the amount of ROS released in the cells increased significantly. In comparison with the model group, no significant difference in cell proliferation rate was found between the caffeic acid groups at different concentrations. This result indicates that 100 *μ*mol/L caffeic acid exerted no protective effect on cells with oxidative damage for 24 h.

When the Mv and Mv3C concentrations were 50 and 100 *μ*mol/L, the cell proliferation rate significantly increased compared with that in the model group, and the intracellular ROS release significantly reduced in a concentration-dependent manner. Compared with that in the Mv group, the cell proliferation rate in the Mv3C group was significantly different, but no statistical difference in ROS level was found. This result suggests that Mv3C not only protects against H_2_O_2_-induced HUVEC cell oxidative damage but also exerts better antioxidant activity than Mv. After the treatment of different concentrations of Mv3G and Mv3CG, when the M3G concentration was 100 *μ*mol/L, the cell proliferation rate significantly increased compared with the model group, and the intracellular ROS release significantly reduced. When the Mv3CG concentrations were 50 and 100 *μ*mol/L, the cell proliferation rate significantly increased compared with that in the model group, and the intracellular ROS release significantly reduced. Compared with those in the Mv3G group, the cell proliferation rate and ROS activity in the Mv3G group exerted no statistical difference. These results indicate that Mv3G and Mv3G exert similar effects. After treatment of the Mv35 G and Mv3C5G groups with different concentrations, the trend was consistent with the above results. In summary, Mv and Mv3C demonstrated the best protective effects and thus were selected to act on the AMPK/SIRT1 signaling pathway, and the antioxidative mechanism was discussed.

### 3.4. Regulation Effect of Mv and Mv3C on the AMPK/SIRT1 Signaling Pathway

#### 3.4.1. Determination of SOD Activity

**Figure 5:**
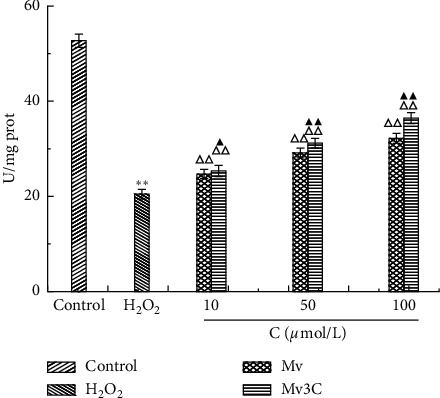
Effects of different concentrations of Mv and Mv3C on level of SOD of HUVEC cells. ^*∗*^*p* < 0.05 and ^*∗∗*^*p* < 0.01 compared to control; Δ*p* < 0.05 and ΔΔ*p* < 0.01 compared to H_2_O_2_; ▲*p* < 0.05 and ▲▲*p* < 0.01 compared to malvidin.

The SOD activity of the model control group was significantly reduced to 0.387 times that of the blank group. Compared with the model control group, different concentrations of Mv and Mv3C significantly increased the activity of SOD in cells in a concentration-dependent manner. At a concentration of 100 *μ*mol/L, the SOD activity increased to 32.941 ± 1.96 U/mg prot and 36.477 ± 1.54 U/mg prot, respectively. Compared with the Mv group, different concentrations of Mv3C significantly increased intracellular SOD activity (Figure 5).

#### 3.4.2. Effect of SIRT1 mRNA Transcription Level

**Figure 6:**
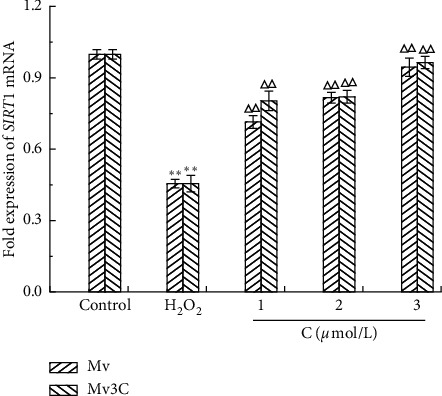
Effects of different concentrations of Mv and Mv3C on expressions of SIRT1 mRNA in HUVEC cells. ^*∗*^*p* < 0.05 and ^*∗∗*^*p* < 0.01 compared to control; Δ*p* < 0.05 and ΔΔ*p* < 0.01 compared to H_2_O_2_; ▲*p* < 0.05 and ▲▲*p* < 0.01 compared to malvidin.

RT-PCR was used to detect the expression level of SIRT1 mRNA in cells. The results are shown in Figure 6. Compared with that in the blank group, the transcription level of SIRT1 mRNA in the model group decreased significantly. After different concentrations of Mv and Mv3C to intervene in damaged cells, the expression level of SIRT1 mRNA in the Mv and Mv3C groups significantly increased compared with that in the model group, and the effect of Mv and Mv3C was similar.

#### 3.4.3. Effects of SIRT1, AMPK, and *p*-AMPK Protein Expression Levels

**Figure 7:**
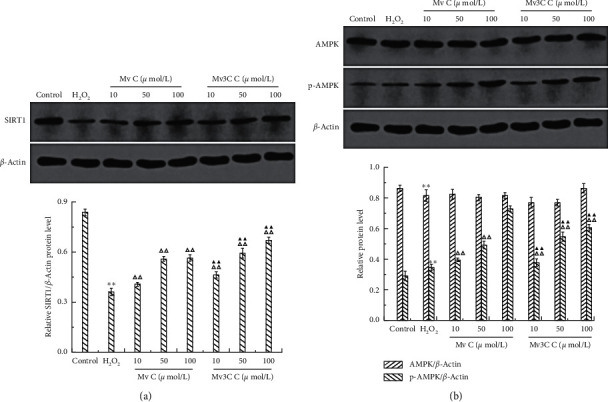
Effect of different concentrations of Mv and Mv3C on the protein expression of SIRT1, *p*-AMPK, and AMPK in HUVEC cells.  ^*∗*^*p* < 0.05 and  ^*∗∗*^*p* < 0.01 compared to control; Δ*p* < 0.05 and ΔΔ*p* < 0.01 compared to H_2_O_2_; ▲*p* < 0.01 and ▲▲*p* < 0.01 compared to malvidin.

Western blot was used to detect the effects of Mv and Mv3C on the expression of SIRT1, AMPK, and *p*-AMPK in cells (Figure 7). Compared with that in the blank group, the expression level of SIRT1 protein in the model group cells decreased significantly, whereas the expression levels of AMPK and *p*-AMPK protein increased slightly. Compared with the model group, the Mv and Mv3C groups at different concentrations significantly increased the expression levels of SIRT1 and *p*-AMPK. They both showed the best effect at 100 *μ*mol/L. The SIRT1 gene was knocked down by siRNA gene fragments for verification to confirm the role of the AMPK/SIRT1 signaling pathway in reducing oxidative stress damage by Mv and Mv3C. Mv and Mv3C at 100 *μ*mol/L were selected to intervene in siRNA-SIRT1-damaged cells. The expression levels of SIRT1, AMPK, and *p*-AMPK in the cells were measured using Western blot.

#### 3.4.4. Screening of Interfering Gene Fragments of Silencing SIRT1

**Figure 8:**
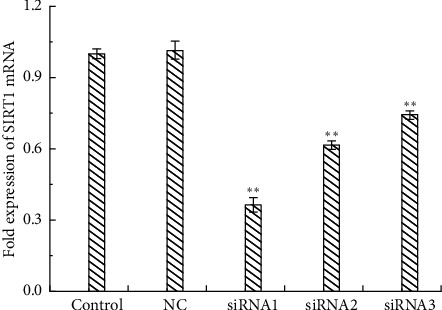
Effect of SiRNA on the expression of SIRT1.  ^*∗*^*p* < 0.05 and  ^*∗∗*^*p* < 0.01 compared to control.

Three SiRNAs and negative control (NC) fragments were designed and synthesized to transfect the cells, and the fragment with the best interference effect on the expression level of SIRT1 mRNA by RT-PCR was selected to silence SIRT1. Compared with the blank control group, the NC group caused no effect on the expression level of SIRT1 mRNA in the cells, and the effect of small exogenous RNA fragments on the experiment could be excluded. Compared with the blank group, all three SiRNAs can significantly reduce the expression level of SIRT1 mRNA, and SiRNA1 decreased to 0.363 (Figure 8). Therefore, SiRNA1 was chosen as the interfering fragment to silence SIRT1.

#### 3.4.5. Effects of SIRT1, AMPK, and *p*-AMPK Protein Expression Levels after Silencing SIRT1

**Figure 9:**
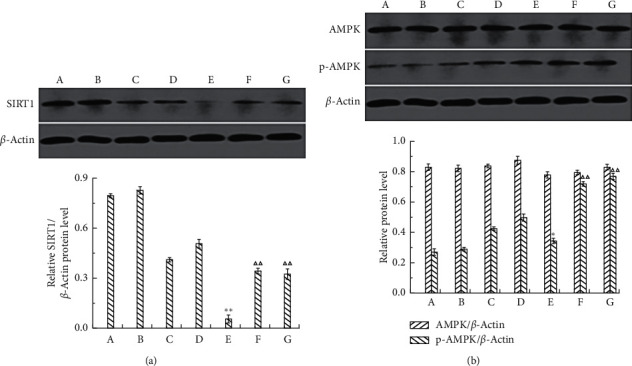
Effects of Mv and Mv3C on the expression of SIRT1, *p*-AMPK, and AMPK after silencing SIRT1. (A) Control, (B) negative control (NC), (C) SiRNA, (D) NC group after H_2_O_2_ induction (H_2_O_2_-NC), (E) SiRNA group after H_2_O_2_ induction (H_2_O_2_-SiRNA), (F) Mv intervention in H_2_O_2_-SiRNA (Mv-H_2_O_2_-SiRNA), and (G) Mv3C intervention in H_2_O_2_-SiRNA (Mv3G-H2O2-SiRNA).  ^*∗*^*p* < 0.05 and  ^*∗∗*^*p* < 0.01 compared to SiRNA. Δ*p* < 0.05 and ΔΔ*p* < 0.01 compared to H_2_O_2_-SiRNA.

As for the influence of SIRT1 protein expression, the SIRT1 protein expression in the SiRNA group significantly reduced compared with that in the blank group and the NC group. Compared with that in the SiRNA group, the expression of SIRT1 protein in the H_2_O_2_-SiRNA group significantly reduced after H_2_O_2_ stimulation. Compared with the H_2_O_2_-SiRNA group, the Mv and Mv3C groups increased SIRT1 protein expression, but the promotion effect was significantly reduced. As for the influence of AMPK and *p*-AMPK protein expression, the expression level of AMPK protein showed no significant change in each group. Compared with the blank group and the NC group, the SiRNA group showed no significant difference in the expression of the *p*-AMPK protein. Compared with that in the SiRNA group, the *p*-AMPK protein expression in the H_2_O_2_-SiRNA group increased slightly, but the change was not significant. Compared with the H_2_O_2_-SiRNA group, the Mv and Mv3C groups increased *p*-AMPK protein expression significantly, but the promotion effect was significantly reduced (Figure 9).

## 4. Discussion

Natural anthocyanins have significant antioxidant activity, but their stability is poor. Ultrahigh pressure can promote the mutual color of anthocyanins and organic acids in the grapevine in a short time, and the stabilizing effect is better than spontaneous secondary color [13]. Mallow pigment was bonded to caffeic acid using ultrahigh pressure technology. The acrylic group was removed first, and then the phenol ring was combined to obtain the bonded product, which was identified as mallow-3-O-guaiacol. Mallow-3-O-glucoside was bonded to caffeic acid, and one molecule of H_2_O was removed to obtain the bonded product, which was identified as mallow-3-O-(6″-O-coffee acyl)-glucoside. When mallow-3,5-O-glucoside was bonded to caffeic acid, one molecule of H_2_O was removed to obtain the bonded product, which was identified as mallow-3-O-(6″-O-coffee acyl)-5-diglucoside.

Anthocyanin compounds are flavonoids whose free radical scavenging activity depends on the difficulty of hydrogen extraction reaction between phenolic hydroxyl groups and free radicals on the ring and the stability of free radicals generated after hydrogen extraction. Therefore, the number and position of phenolic hydroxyl groups affect the antioxidant effect of anthocyanins. The structure of the C-ring greatly influences the activity of flavonoids. C_3(2)_-OH, C_2_=C_3_ double bond, and C4 carbonyl on C-ring can enhance the activity of flavonoids. The phenolic hydroxyl group at the C4′ position in the B ring is the main active site; the phenolic hydroxyl groups at C4′ and C3 positions are active. The phenolic hydroxyl group at the C5 position on the A ring is also active [28, 29]. The results of this study prove the conclusions of the above scholars. Mv, Mv3G, Mv35 G, Mv3C, Mv3CG, and Mv3C5G all have phenolic hydroxyl groups at the C4′ position, which can significantly increase the proliferation rate of injured cells and reduce intracellular ROS release. Phenolic hydroxyl groups are present at the C5 position in the Mv and Mv3C structures, and their activities are greater than those of Mv3CG, Mv3C5G, Mv3G, and Mv35 G. This finding proves that the C5 phenolic hydroxyl activity in the above literature is also strong. The activity of the stable compound Mv3C was significantly better than that of the untreated Mv because the O at the C3 position in the Mv3C structure is linked to catechol, with many phenolic hydroxyl groups and strong activity.

Recent studies have shown that the AMPK/SIRT1 signaling pathway is closely related to aging because of oxidative stress and its related diseases [30]. AMPK is the highly conservative cellular energy metabolism receptor and regulator. During oxidative stress, AMPK protein expression or activity decreases [31]. SIRT1 is closely related to physiological processes, such as cell proliferation, differentiation, senescence, apoptosis, and metabolism. SIRT1 can be used as a key regulatory point in oxidative stress to reduce cell damage [32]. In the present study, Mv and Mv3C significantly increased the expression of SIRT1 mRNA and SIRT1, *p*-AMPK protein after oxidative damage to HUVEC cells, indicating that Mv and Mv3C can promote the phosphorylation of AMPK and upregulate the expression of SIRT1 mRNA and SIRT1 protein. This result suggests that stabilization of malvid anthocyanins play an antioxidative role in activating the AMPK/SIRT1 signaling pathway. SiRNA was added to silence SIRT1 gene expression and then the injured cells were treated with Mv and Mv3C to confirm the role of the AMPK/SIRT1 signaling pathway in Mv and Mv3C in reducing oxidative stress damage. This procedure significantly inhibited the effect of Mv and Mv3C in upregulating SIRT1 and *p*-AMPK protein expression. This result proves that the AMPK/SIRT1 signaling pathway is important in the antioxidative role of stabilization of malvid anthocyanins.

## 5. Conclusion

This study is the first to analyze the antioxidant activity of the binding products of malvid anthocyanins and caffeic acid. The protective effect of stabilization of malvid anthocyanins on H_2_O_2_-induced oxidative damage on HUVECs was studied. The anthocyanins significantly increased cell proliferation rate, inhibited ROS production, increased intracellular SOD activity, and upregulated the expression levels of SIRT1 mRNA and SIRT1, *p*-AMPK protein. The addition of siRNA to silence SIRT1 gene expression significantly suppressed the effect of Mv and Mv3C in upregulating protein expression. The AMPK/SIRT1 signaling pathway plays an important role in the antioxidative role of the stabilization of malvid anthocyanins. This study provides a basis for the subsequent product development anthocyanins.

## Figures and Tables

**Table 1:** MS/MS data of the bonded products.

No.	Molecular formula	Calculated (*m/z*)	Measured (*m/z*)	Fragmention (*m/z*)	Relative error (10^−6^)	Component name
M1	C_23_H_19_O_9_	439.10291	439.10531	439.10351	1.37	Malvidin-3-O-guaiacol
				331.12247		
				150.33725		

M2	C_29_H_35_O_17_	655.16630	655.16632	655.16632	0.03	Malvidin-3-O-(6″-O-caffeoyl)-glucoside
				493.17451		
				331.14216		
				150.38797		

M3	C_38_H_41_O_20_	817.21912	817.21914	817.21914	0.02	Malvidin-3-O-(6″-O-caffeoyl)-5-diglucoside
				655.34326		
				493.13761		
				331.12026		
				150.38903		

**Table 2:** Effect of hydrogen peroxide on cell semi-inhibition at different time points.

Group	6 h	12 h	24 h	36 h
IC_50_(*μ*mol/L)	701.643	616.683	385.778	301.644

**Table 3:** Effects of different concentrations of H_2_O_2_ on ROS in cells after different time points.

C (*μ*mol/L)	R			
	6 h	12 h	24 h	48 h
0	1.00	1.00	1.00	1.00
100	1.06	1.10	1.05	1.06
200	1.12	1.23	1.35^*∗*^	1.37^*∗*^
300	1.24^*∗*^	1.35^*∗*^	2.56^*∗∗*^	2.57^*∗∗*^
500	2.22^*∗∗*^	1.83^*∗∗*^	2.25^*∗∗*^	2.01^*∗∗*^
750	3.24^*∗∗*^	2.01^*∗∗*^	1.79^*∗∗*^	1.80^*∗∗*^
1000	2.86^*∗∗*^	2.73^*∗∗*^	1.12^*∗∗*^	0.97

^*∗*^*P* < 0.05 and ^*∗∗*^*P* < 0.01 compared to control (0 *μ*mol/L).

## Data Availability

The data that support the findings of this study are available from the corresponding author, L. -K. W., upon reasonable request.

## References

[1] Gohar U. F., Iqbal I., Shah Z., Mukhtar H., Zia-Ul-Haq M., Zia-Ul-Haq M., Bin-Jumah M. N., Alothamn S. I., Henidi H. A. (2021). COVID-19: recent developments in therapeutic approaches. *Alternative Medicine Interventions for COVID-19*.

[2] Zia-Ul-Haq M., Zia-Ul-Haq M., Dewanjee S., Riaz M. (2021). Historical and introductory aspects of carotenoids. *Carotenoids: Structure and Function in the Human Body*.

[3] Zhang J., Zhang S. D., Wang P (2019). Pinolenic acid ameliorates oleic acid-induced lipogenesis and oxidative stress via AMPK/SIRT1 signaling pathway in HepG2 cells. *European Journal of Pharmacology*.

[4] Morsch A. L. B. C., Wisniewski E., Luciano T. F (2019). Cigarette smoke exposure induces ROS-mediated autophagy by regulating sestrin, AMPK, and mTOR level in mice. *Redox Report*.

[5] Moroz N., Carmona J. J., Anderson E., Hart A. C., Sinclair D. A., Blackwell T. K. (2014). Dietary restriction involves NAD + ‐dependent mechanisms and a shift toward oxidative metabolism. *Aging Cell*.

[6] Sung B., Chung J. W., Bae H. R. (2015). Humulus japonicus extract exhibits antioxidative and anti-aging effects via modulation of the AMPK-SIRT1 pathway. *Experimental and Therapeutic Medicine*.

[7] Li Y., Xu S., Mihaylova M. M. (2011). AMPK phosphorylates and inhibits SREBP activity to attenuate hepatic steatosis and atherosclerosis in diet-induced insulin-resistant mice. *Cell Metabolism*.

[8] Huang C. H., Shiu S. M., Wu M. T. (2013). Monacolin K affects lipid metabolism through SIRT1/AMPK pathway in HepG2 cells. *Archives of Pharmacal Research*.

[9] Xiuyun H., Shanqin X., Maitland-Toolan K. A. (2008). SIRT1 regulates hepatocyte lipid metabolism through activating AMP-activated protein kinase. *Journal of Biological Chemistry*.

[10] Jeon B. T., Jeong E. A., Shin H. J. (2012). Resveratrol attenuates obesityassociated peripheral and central inflmmation and improves memory defiit in mice fed a high-fat diet. *Diabetes*.

[11] Wen L. K., He Y., Du Y. J. (2016). Analysis of anthocyanins content different varieties of Vitis amurensis. *Oxidation Communications*.

[12] Huang W. Y., WanG J., Liu Y. M. (2014). Inhibitory effect of malvidin on TNF-*α*-inducedinflammatory response in endothelial cells. *European Journal of Pharmacology*.

[13] He Y., Wen L. K., Yu H. (2018). Effects of high hydrostatic pressure-assisted organic acids on the copigmentation of Vitis amurensis Rupr anthocyanins. *Food Chemistry*.

[14] He Y., Wen L. K., Liu J. S. (2017). Optimisation of pulsed electric fields extraction of anthocyanin from Beibinghong Vitis Amurensis Rupr. *Natural Product Research*.

[15] Oliveira M. C., Esperança P., Almoster Ferreira M. A. (2001). Characterisation of anthocyanidins by electrospray ionisation and collision-induced dissociation tandem mass spectrometry. *Rapid Communications in Mass Spectrometry*.

[16] Zou B., Xu Y. J., Wu J. J. (2017). Phenolic compounds participating in mulberry juice sediment formation during storage. *Journal of Zhejiang University Science B*.

[17] Hidalgo M., Martin-Santamaria S., Recio I. (2012). Potential anti-inflammatory, anti-adhesive, anti/estrogenic, and angiotensin-converting enzyme inhibitory activities of anthocyanins and their gut metabolites. *Genes & Nutrition*.

[18] Schön C., Wacker R., Micka A. (2018). Bioavailability study of maqui berry extract in healthy subjects. *Nutrient*.

[19] Czank C., Cassidy A., Zhang Q. (2013). Human metabolism and elimination of the anthocyanin, cyanidin-3-glucoside: a 13C-tracer study. *The American Journal of Clinical Nutrition*.

[20] Fornasaro S., Ziberna L., Gasperotti M. (2016). Determination of cyanidin 3-glucoside in rat brain, liver and kidneys by UPLC/MS-MS and its application to a short-term pharmacokinetic study. *Scientific Reports*.

[21] Cao G., Muccitelli H. U., Sánchez-Moreno C. (2001). Anthocyanins are absorbed in glycated forms in elderly women: a pharmacokinetic study. *The American Journal of Clinical Nutrition*.

[22] Vitaglione P., Donnarumma G., Napolitano A. (2007). Protocatechuic acid is the major human metabolite of cyanidin-glucosides. *Journal of Nutrition*.

[23] Montoro P., Tuberoso C. I., Perrone A. (2006). Characterisation by liquid chromatography-electrospray tandem mass spectrometry of anthocyanins in extracts of Myrtus communis L. berries used for the preparation of myrtle liqueur. *Journal of Chromatography A*.

[24] de Rosso V. V., Mercadante A. Z. (2007). HPLC-PDA-MS/MS of anthocyanins and carotenoids from dovyalis and tamarillo fruits. *Journal of Agricultural and Food Chemistry*.

[25] Forester S. C., Waterhouse A. L. (2010). Gut metabolites of anthocyanins, gallic acid, 3-O-methylgallic acid, and 2,4,6-trihydroxybenzaldehyde, inhibit cell proliferation of Caco-2 cells. *Journal of Agricultural and Food Chemistry*.

[26] De Ferrars R. M., Cassidy A., Curtis P. (2014). Phenolic metabolites of anthocyanins following a dietary intervention study in post-menopausal women. *Molecular Nutrition & Food Research*.

[27] Wu X., Prior R. L. (2005). Systematic identification and characterization of anthocyanins by HPLC-ESI-MS/MS in common foods in the United States: fruits and berries. *Journal of Agricultural and Food Chemistry*.

[28] Fang J. G., Zhou B. (2008). Structure-activity relationship and mechanism of the tocopherol regenerating activity of resveratrol and its analogues. *Journal of Agricultural and Food Chemistry*.

[29] Farhadi F., Khameneh B., Iranshahi M., Iranshahy M. (2019). Antibacterial activity of flavonoids and their structure-activity relationship: an update review. *Phytotherapy Research*.

[30] Gao Q. (2019). Oxidative stress and autophagy. *Advances in Experimental Medicine and Biology*.

[31] Hardie D. G., Ross F. A., Hawley S. A. (2012). AMPK: a nutrient and energy sensor that maintains energy homeostasis. *Nature Reviews Molecular Cell Biology*.

[32] Ou X., Lee M. R., Huang X. (2014). SIRT1 positively regulates autophagy and mitochondria function in embryonic stem cells under oxidative stress. *Stem Cells*.

